# Differential recruitment of ventral pallidal e-types by behaviorally salient stimuli during Pavlovian conditioning

**DOI:** 10.1016/j.isci.2021.102377

**Published:** 2021-03-31

**Authors:** Panna Hegedüs, Julia Heckenast, Balázs Hangya

**Affiliations:** 1 Lendület Laboratory of Systems Neuroscience, Institute of Experimental Medicine, Budapest 1083, Hungary; 2 János Szentágothai Doctoral School of Neurosciences, Semmelweis University, Budapest 1085, Hungary

**Keywords:** Cellular Physiology, Behavioral Neuroscience, Cellular Neuroscience

## Abstract

The ventral pallidum (VP) is interfacing striatopallidal and limbic circuits, conveying information about salience and valence crucial to adjusting behavior. However, how VP neuron populations with distinct electrophysiological properties (e-types) represent these variables is not fully understood. Therefore, we trained mice on probabilistic Pavlovian conditioning while recording the activity of VP neurons. Many VP neurons responded to punishment (54%), reward (48%), and outcome-predicting auditory stimuli (32%), increasingly differentiating distinct outcome probabilities through learning. We identified e-types based on the presence of bursts or fast rhythmic discharges and found that non-bursting, non-rhythmic neurons were the most sensitive to reward and punishment. Some neurons exhibited distinct responses of their bursts and single spikes, suggesting a multiplexed coding scheme in the VP. Finally, we demonstrate synchronously firing neuron assemblies, particularly responsive to reinforcing stimuli. These results suggest that electrophysiologically defined e-types of the VP differentially participate in transmitting reinforcement signals during learning.

## Highlights

• VP neurons are sensitive to reward, punishment, and expectation during conditioning
• Non-bursting, non-rhythmic VP neurons respond more often to salient events
• VP neurons form synchronously firing assemblies responsive to reinforcement
• Multiplexed single spike and burst codes were found in the VP

## Graphical abstract

**Figure:**
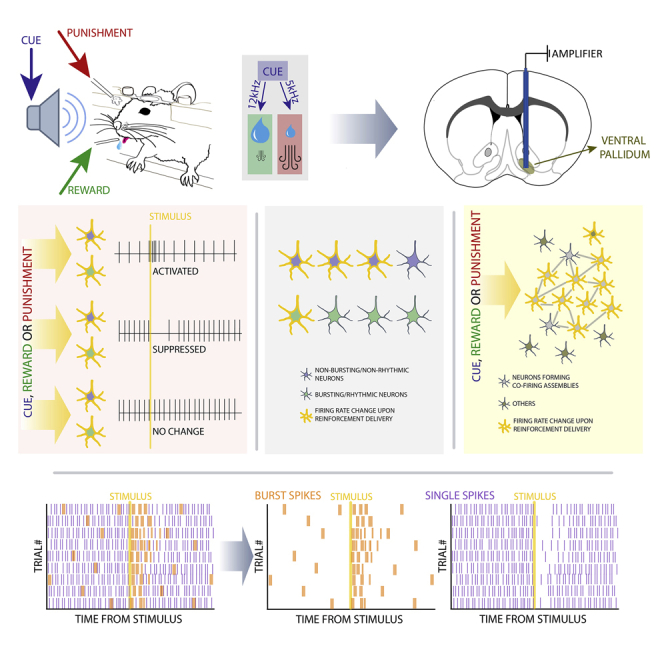

## Introduction

The ventral pallidum (VP) serves as an interface between the limbic system and other structures, integrating cortical, amygdala, basal ganglia, and neuromodulatory input (Root et al., 2015; Záborszky and Cullinan, 1992). On the effector side, its projections to the thalamus, cortex, basal ganglia, and other subcortical structures including hypothalamus, ventral tegmental area (VTA), and lateral habenula (LHb) influence motivation, reinforcement learning, and attention (Faget et al., 2018; Ito and Doya, 2009; Maurice et al., 1997; Richard et al., 2016; Root et al., 2015; Smith et al., 2009; Stephenson-Jones et al., 2020; Zahm et al., 1996). Specifically, crucial aspects of VP activity in associative learning have been revealed recently, showing VP neurons encoding incentive salience and valence as well as mounting behavioral response to environmental changes (Avila and Lin, 2014; Richard et al., 2016; Stephenson-Jones et al., 2020; Tindell, 2004; Tindell et al., 2006).

How are different types of information encoded by the VP? Whether they are routed through different lines of this intricate switch board, labeled by markers such as parvalbumin, the vesicular glutamate transporter VGluT2, or the inhibitory marker GAD2 has been explored recently (Faget et al., 2018; Knowland et al., 2017; Prasad et al., 2020; Stephenson-Jones et al., 2020; Wulff et al., 2019). However, another exciting possibility is that integrating and multiplexing is also represented by different coding schemes including elements of rate and temporal code, such as characteristic firing patterns like bursts or single spike firing, rhythmic discharges, network level synchrony, and asynchronous activity (Ascoli et al., 2008; Gouwens et al., 2019; de Vries et al., 2020). Accordingly, Avila and Lin suggest that electrophysiological characterization that goes beyond the broad categories of inhibitory and excitatory cell types will enable a better understanding of how VP performs its functions (Avila and Lin, 2014).

To address the aforementioned question, we recorded VP neurons while mice performed a probabilistic Pavlovian conditioning task. By using auto- and cross-correlation techniques, we uncovered the presence of separate fast rhythmic, bursting, and non-bursting-non-rhythmic neurons, similar to previous electrophysiological categorization of VP neurons (Pang et al., 1998). We found that reinforcement-related signals were most frequent in the non-bursting, non-rhythmic population. The analysis of synchronous discharges revealed the presence of co-firing neuron assemblies. Cells that participated in these synchronously firing assemblies showed increased responsiveness to reinforcers. Thus, VP neurons with distinct discharge types (“e-types”) both at the individual and network level, likely corresponding to different coding strategies, show differences in their representation of behaviorally salient stimuli. Even within single neurons, burst and single spike firing could strongly dissociate, suggesting the presence of a distinct burst code in the VP (Kepecs and Lisman, 2003; Kepecs et al., 2002).

## Results

### Ventral pallidal neurons are sensitive to reward, punishment, and expectation during Pavlovian conditioning

**Figure 1:**
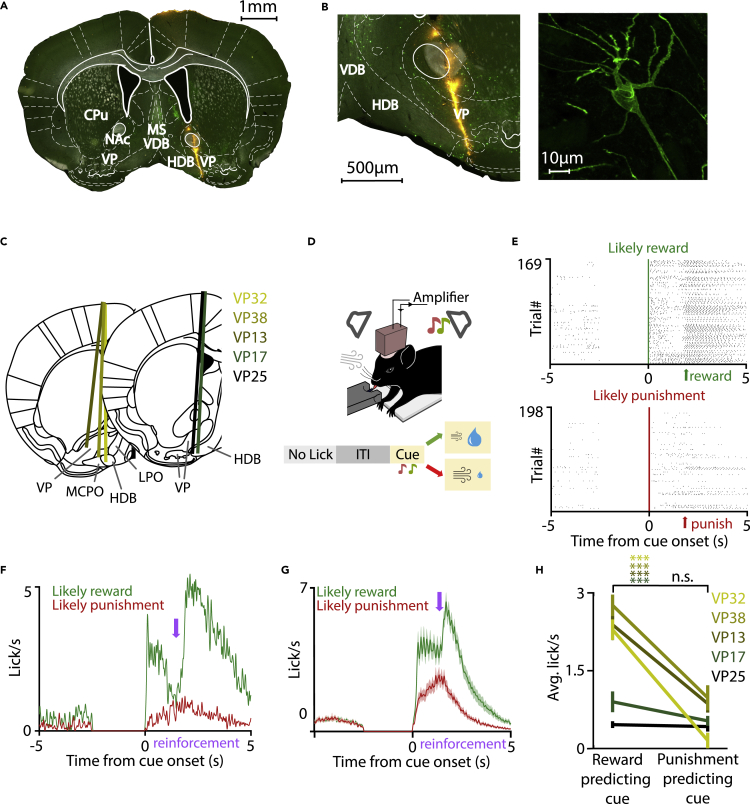
Targeting VP in mice performing auditory Pavlovian conditioning (A) Coronal section from a ChAT-Cre mouse showing the tetrode tracks (DiI, yellow; ChAT+, green) through the VP. The tetrodes were advanced 0–100 μm between recording days. Although the images show the full extent of the electrode tracks, only those sessions that were conducted strictly within VP boundaries based on post hoc histological reconstruction (see transparent methods) were included. Scale bar, 1 mm. (B) Left, magnified view of the target area. Scale bar, 500 μm. Right, confocal image of a cholinergic neuron located near the electrode track (20× magnification, z stack of 8 planes, maximal intensity projection). Scale bar, 10 μm. (C) Reconstructed location of the electrode tracks. Only neurons recorded inside the VP were included (see transparent methods, histology). (D) Top, schematic of the auditory Pavlovian task setup. Bottom, trial structure with possible outcomes. After the mouse stopped collecting the previous reward (“no lick”), a variable inter-trial interval started, signaled by turning an light-emitting diode off, in which no licking was allowed. Then two cue tones of well-separated pitch predicted likely reward or likely punishment. (E) Raster plots of licking activity in an example session of one mouse. The cue predicting likely reward (top, green) elicited stronger anticipatory licking than the cue that signaled likely punishment (bottom, red). (F) Peri-event time histograms (PETH) of licking activity from the same session. Purple arrow indicates average reinforcement delivery time (note that reinforcement time was randomized between 400 and 600 ms after cue offset according to a uniform distribution to prevent full temporal predictability of reinforcement delivery). (G) Average licking activity (PETH) of N = 5 mice shows stronger anticipatory licking after the cue that predicted likely reward (green). Data are represented as mean ± SEM. (H) The reward-predicting cue elicited significantly more licks in 4/5 mice. Data are represented as median ±SE of median. ∗∗∗p < 0.001, Wilcoxon signed rank test. CPu, caudate putamen; HDB, horizontal nucleus of the diagonal band of Broca; MS, medial septum; NAc, nucleus accumbens; VDB, vertical nucleus of the diagonal band of Broca.

To test how different VP neurons represent behaviorally relevant events during classical associative learning, we trained mice (N = 5) on a probabilistic auditory Pavlovian conditioning task and monitored the activity of VP neurons (n = 704) (Figures 1A–1C). Mice were water restricted and head-fixed for training, listening to two pure tones of different pitch, where one tone predicted likely water reward (80% reward, 10% punishment, 10% omission) and the other tone predicted likely punishment (25% reward, 65% punishment, 10% omission; Figure 1D). Mice learned to discriminate the cue tones, indicated by differential licking activity after cue onset in anticipation of reward (Figures 1E–1G). Four of five animals showed significant behavioral discrimination at the individual level (Figure 1H).

**Figure 2:**
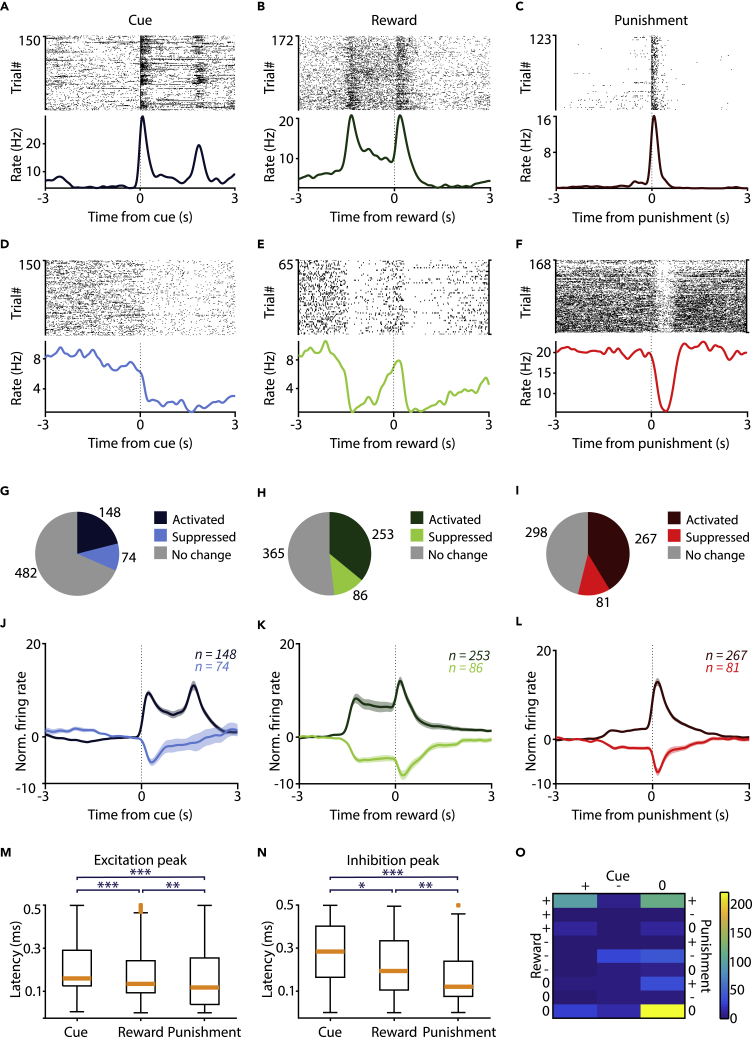
Ventral pallidal neurons are modulated by reward, punishment, and outcome-predictive cues during Pavlovian conditioning (A–F) Example single VP neurons activated by cue stimuli (A), reward (B) or punishment (C), or inhibited by cue (D), reward (E), or punishment (F). Top, spike raster; bottom, PETH aligned to the respective behavioral events. (G–I) Pie charts showing the number of all VP neurons activated and inhibited by cue (G), reward (H), and punishment (I), pooled across mice. (J–L) Average *Z*-scored PETH of all recorded VP neurons modulated by cue (J), reward (K), or punishment (L), tested separately for the three events. Data are represented as mean ± SEM. (M) Excitatory response latencies to predictive cues, reward, and punishment. Box-whisker plots show median, interquartile range, non-outlier range, and outliers. ∗∗p < 0.01; ∗∗∗p < 0.001, Mann-Whitney U test. (N) Inhibitory response latencies to predictive cues, reward, and punishment. Box-whisker plots show median, interquartile range, non-outlier range, and outliers. ∗p < 0.05; ∗∗p < 0.01, ∗∗∗p < 0.001, Mann-Whitney U test. (O) Number of neurons showing different combinations of all possible responses to cues and reinforcers. +, activation; -, suppression; 0, no significant firing rate change. For instance, the color in line 1, column 1 indicates the number of neurons that were activated by all three salient events tested (cue, reward, punishment); color in line 2, column 2 indicates the number of neurons that were activated after reward but inhibited both after sound cues and punishment. See also Figures S1 and S2.

During the task, 66% VP neurons showed phasic, short latency activation or inhibition after at least one type of behaviorally salient stimuli of the task, that is, reward, punishment, and/or the reinforcement-predicting cues (Figures 2A–2F). Around 32% (n = 222/704) of the neurons were modulated by the cues, 48% (n = 339/704) by reward, and 54% (n = 348/646, neurons recorded early in training could not be tested; Mann-Whitney U test, p < 0.001) by punishment (Figures 2G–2L). The majority of significantly responsive neurons showed activation, with a smaller fraction of inhibited cells (activated by cue, 148/222, 67%; reward, 253/339, 75%; punishment, 267/348, 77%). Moreover, the fraction of neurons responding to cue, reward, or punishment tended to correlate with the anticipatory lick rate difference of the animals during the task (Figures S1A–S1C). Interestingly, there was a difference in the latency of peak activation or inhibition across responses to cue, reward, or punishment. VP neurons responded the fastest to punishment, intermediate to reward, and slowest to outcome-predictive sensory cues (Figures 2M–2N).

We found that neural responses to reinforcement of opposite valence were often correlated. For instance, in 222/646 neurons, the same neuron responded with increased firing rate to both the positive and negative reinforcer. Similarly, 70/646 neurons showed firing rate decrease after both reward and punishment (Figure 2O). In contrast, only n = 3/646 neurons showed opposite responses to reward and punishment. Additionally, a large fraction of neurons showed correlated responses to reward-predictive cues and primary reward (n = 109/646 activation and n = 35/646 inhibition to both, respectively). We have found a strong co-occurrence of activation to all behaviorally salient events including cue, reward, and punishment (n = 98/646).

Given that responses to water and air-puff were often correlated, we considered whether air-puff responses could be more related to a lack of reward than the aversive quality of the air-puff. We found this was unlikely, because (1) we had shown that air-puffs were consistently avoided by mice in an operant paradigm (Figure 2C in Hangya et al. [2015]) trained in our setup (Solari et al., 2018), (2) air-puffs were accompanied by different auditory input compared with water reward rendering sensory response generalization unlikely (Figure S2A), and (3) little evidence was found for reward omission responses in the VP (Figures S2B and S2C).

In sum, VP neurons showed an array of responses to behaviorally salient events. The fastest and most prevalent response pattern was a rapid activation after punishment. Direction of firing rate modulation was correlated across behaviorally relevant events, suggesting salience coding by individual VP neurons.

The probabilistic nature of the Pavlovian task meant that different cues were followed by reward, punishment, or omission with different (but fixed) contingencies. Mice learned these contingencies (Figure 1), which required integration of positive and negative outcomes over many trials. Based on the predictive auditory cues we played before the reinforcement, reward and punishment could be either expected or surprising according to task contingencies. This allowed us to compare VP neuronal responses with expected versus surprising outcomes.

**Figure 3:**
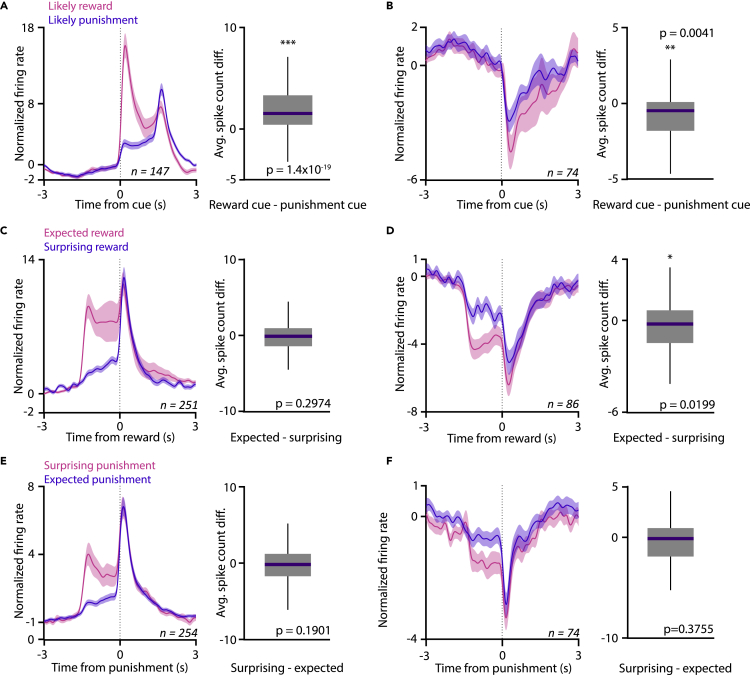
Ventral pallidal neurons are modulated by expectation (A) Left, average PETH of cue-activated VP neurons after cues predicting likely reward (pink) or likely punishment (purple). Right, box-whisker plot of average spike count difference between the two outcome probability conditions in VP neurons activated by cue presentations. Data are represented as mean ± SEM. ∗∗∗p < 0.001, Wilcoxon signed rank test. (B) Average PETH and spike count difference for VP neurons inhibited after cue presentations. Data are represented as mean ± SEM. ∗∗p < 0.01, Wilcoxon signed rank test. (C and D) Same as in (A and B), but showing responses to reward presentations. Left, reward-activated VP neurons; right, reward-suppressed VP neurons. Pink, expected reward (reward delivery after the likely reward cue); purple, surprising reward (reward delivery after the likely punishment cue). Data are represented as mean ± SEM. ∗p < 0.05, Wilcoxon signed rank test. (E and F) Same as in (A and B), but showing responses to punishment presentations. Left, punishment-activated VP neurons; right, punishment-suppressed VP neurons. Pink, surprising punishment (punishment delivery after the likely reward cue); purple, expected punishment (punishment delivery after the likely punishment cue). Data are represented as mean ± SEM. Recordings with less than 5 trials in either of the tested conditions were excluded. Box-whisker pots show median, interquartile range, and non-outlier range in all panels. See also Figures S1 and S2. See also Figure S1 and S2.

We found that VP neurons strongly differentiated the distinct predictive cues, showing larger responses to cues that predicted likely reward (Figures 3A and 3B), reminiscent of prediction error coding (Kim et al., 2020; Schultz et al., 1997). This differential activity was more prominent in VP neurons recorded from mice that exhibited stronger behavioral discrimination of the predictive cues (Figure S3). Although reinforcement error models predict a smaller response to expected compared with surprising reward, we did not observe a significant difference (Figures 3C–3F). This might be due to the relatively small percentage of true reward prediction error-coding VP neurons that might prevent the detection of potentially small expectation-driven differences of reward responses. These results are consistent with a hypothesized role of the VP in signaling incentive salience (Ahrens et al., 2016, 2018; Tindell et al., 2005) and partially support recent findings indicating prediction error coding in the VP (Ottenheimer et al., 2020).

### Non-bursting, non-rhythmic VP neurons are more often recruited by behaviorally salient events

**Figure 4:**
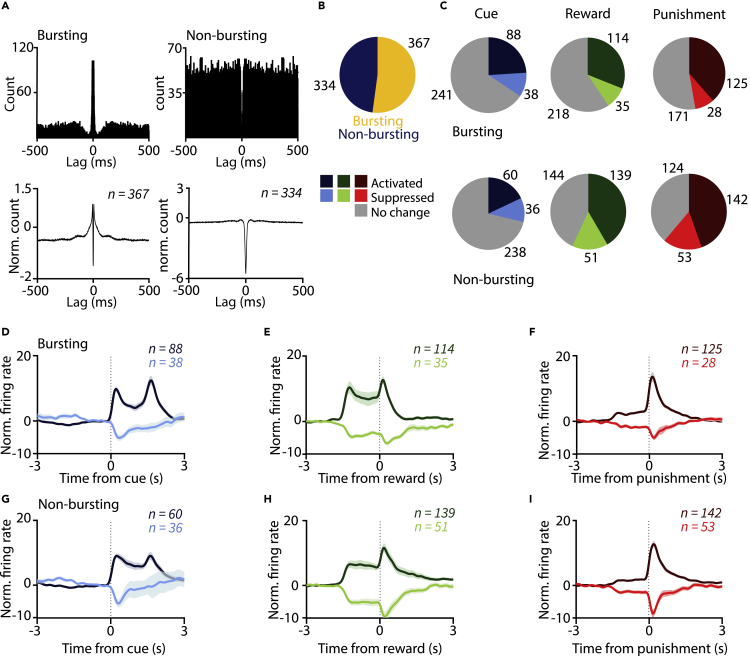
Non-bursting ventral pallidal neurons respond to reinforcers more frequently (A) Top, Example autocorrelograms of a single bursting and a non-bursting VP neuron. Bottom, average across all bursting and non-bursting neurons recorded from the VP. (B) Pie chart showing the proportion of bursting and non-bursting neurons, pooled across mice. (C) Pie charts showing the number of all bursting and non-bursting VP neurons activated and inhibited by cue, reward, or punishment. (D–I) Average, *Z*-scored PETHs of bursting (D–F) and non-bursting (G–I) VP neurons aligned to cue (D and G), reward (E and H), and punishment (F and I). Data are represented as mean ± SEM. See also Figures S4–S6.

Burst coding of salient events has emerged as a general scheme for subcortical representations: burst responses to behavioral reinforcement and reward-predictive stimuli has been demonstrated for the VTA (Schultz et al., 1997), striatum, basal forebrain (Hangya et al., 2015; Lin and Nicolelis, 2008), and LHb (Yang et al., 2018). To test whether this principle generalizes to the VP, we categorized VP neurons as bursting and non-bursting based on short-latency (<10 ms) peaks in their spike autocorrelations, indicating preferential firing with short inter-spike intervals characteristic of bursts (Laszlovszky et al., 2020; Royer et al., 2012) (Figure 4A; transparent methods).

We found that about half of VP neurons (n = 367/701, 52%; 3 neurons firing <100 spikes excluded from this analysis) fired bursts, whereas the remaining neurons were categorized as non-bursting (n = 334/701, 48%, Figure 4B). Next, we tested whether bursting neurons were more responsive to reward, punishment, and reward-predictive cues in Pavlovian conditioning. Surprisingly, we found that a larger fraction of non-bursting VP neurons showed significant responses to reinforcement (chi-square test, p = 1.64 × 10^−5^ and p = 1.01 × 10^−5^ for reward and punishment, respectively, Figures 4C–4I). This was consistent for both reward and punishment, with a higher number of non-bursting neurons showing either firing rate increase or decrease. We did not find any difference regarding the fraction of cue-responsive neurons (chi-square test, p = 0.1121). These findings were largely consistent across animals included in the experiment (Figure S4) and did not depend on the interspike interval cutoff used for defining bursts in extracellular recordings (Figure S5). Response magnitudes were variable across neurons; we provide balanced averages in Figures 4D–4I; however, *Z* score normalization conceals response magnitude variations in these plots. We directly compared response magnitudes across bursting and non-bursting neurons and found that activation after predictive cues or punishment was significantly larger in non-bursting neurons at a conservative p < 0.01 significance threshold (p = 0.005 and p = 0.001, respectively; Mann-Whitney U-test). In addition, activation or inhibition following reward and inhibition after punishment were marginally larger in non-bursters (p = 0.017, p = 0.053, p = 0.014, respectively; Mann-Whitney U test). Non-bursting neurons also showed higher baseline firing rates (Figure S6A).

**Figure 5:**
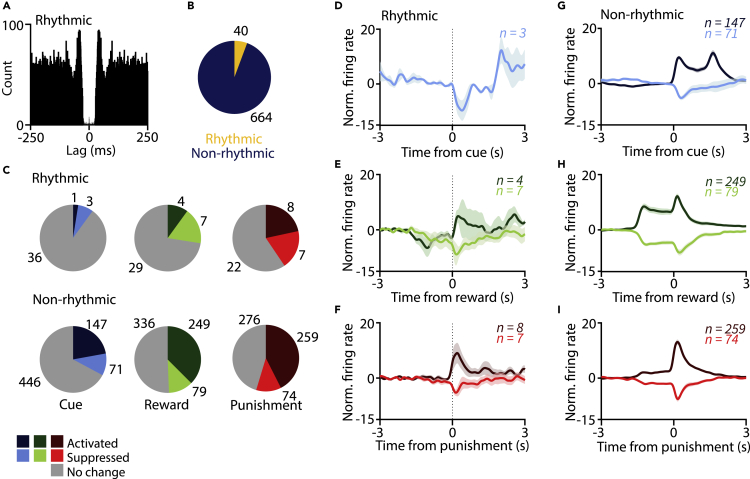
Salient events mostly recruit non-rhythmic neurons (A) Example autocorrelogram of a rhythmically firing neuron. (B) Pie chart showing the proportion of rhythmic and non-rhythmic neurons, pooled across mice. (C) Pie charts showing the number of rhythmic and non-rhythmic VP neurons activated or inhibited by cue, reward, or punishment, pooled across mice. (D–I) Average, *Z*-scored PETHs of rhythmic (D–F) and non-non-rhythmic (G–I) VP neurons aligned to cue (D and G), reward (E and H), and punishment (F and I). Data are represented as mean ± SEM. See also Figure S7.

Based on rhythmic modulation of their autocorrelation functions, we detected a small subset of rhythmically firing VP neurons (n = 40/704, 6%; Figures 5A and 5B). We estimated the frequency at which these neurons were oscillating by their autocorrelation peak location and found that they fell in the beta/gamma range (6 beta-rhythmic and 34 gamma-rhythmic neurons were detected; see transparent methods). These neurons have previously been identified as somatostatin-expressing GABAergic neurons (Espinosa et al., 2019). We found that most of these rhythmically discharging neurons showed weak or no responses to behaviorally salient events including cue tones, reward, and punishment (Figures 5C–5I; largely consistent across mice, see Figure S7). These results suggest that mostly non-bursting, non-rhythmic neurons are recruited during reinforcement learning in the VP.

### Indications of multiplexed burst and single spike code in the VP

Theoretical studies have suggested that because specific biophysical mechanisms are engaged to serve burst generation, burst firing may carry a representation independent from that of single spikes, creating a specific “burst code” (Kepecs and Lisman, 2003; Kepecs et al., 2002). This may allow neurons to multiplex different sources of information; however, this idea has rarely been tested.

**Figure 6:**
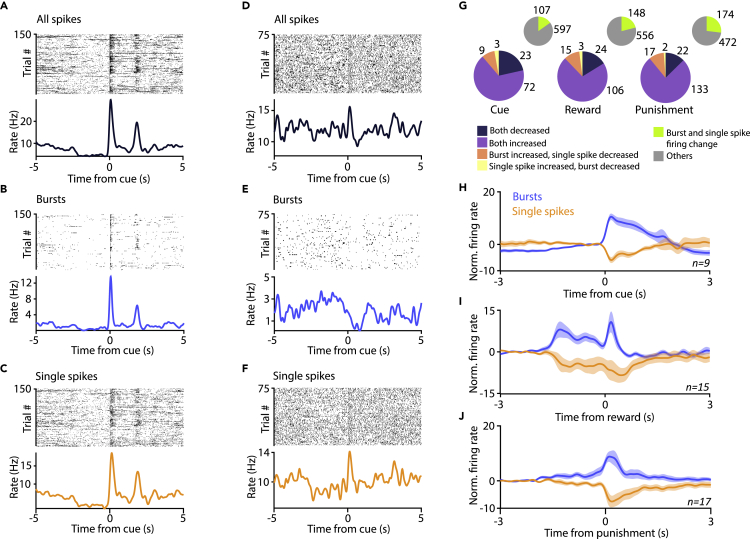
Dissociation of single spike and burst firing in a subset of VP neurons (A) Example PETH of a VP neuron activated upon cue onset. (B and C) The neuron showed increased burst (B) and single spike firing (C) after the cue. (D–F) (D) Example of another cue-activated neuron, which showed decreased burst firing (E) and increased single spike firing (F) after cue presentation. (G) Pie chart showing the number of neurons responding with significant rate change of both bursts and single spikes after cue (left), reward (middle), or punishment (right), pooled across mice. Insert, number of neurons where a significant change was detected for both bursts and single spikes (green). (H–J) Average *Z*-scored PETH of burst and single spike responses of neurons where bursts showed an increase, whereas single spikes showed a decrease after cue (H), reward (I), or punishment (J). Data are represented as mean ± SEM. See also Figure S8.

Therefore, we separated burst firing and single spike firing based on inter-spike interval (ISI) criteria. Specifically, bursts were defined by the first ISI <10 ms and subsequent ISIs <15 ms (Laszlovszky et al., 2020; Royer et al., 2012). Next, bursts and single spikes of each neuron were aligned to behaviorally salient events. Burst and single spike firing often carried similar information about these events, indicated by correlated peri-event time histograms (PETHs) showing similar dynamics for bursts and single spikes (Figures 6A–6C). However, a subset of bursting VP neurons showed a dissociation of burst and single spike coding. The example neuron in Figures 6D–6F (enlarged in Figure S8) increased its firing rate after cue tone presentation. However, analysis of burst and single spike occurrence revealed that whereas single spike firing was elevated after the cues (Figure 6F), burst firing showed a concurrent inhibition (Figure 6E; this was not due to insufficient spike sorting). We quantified the proportion of VP neurons that showed significant opposite change of firing rate when bursts and single spikes were considered (Figures 6G-6J; opposite responses were found in 12/107, 11% of cue-responsive VP neurons; 18/148, 12% of reward-responsive VP neurons; 19/174, 11% of punishment-responsive VP neurons; for reference populations, we used the neurons where significant changes in both single spikes and bursts were found; p < 0.01, Mann-Whitney U test). This suggests that a subset of bursting VP neurons exhibit separate representations of external events by bursts and single spikes, revealing a distinct “burst code.”

### Ventral pallidal neurons form synchronously firing assemblies

**Figure 7:**
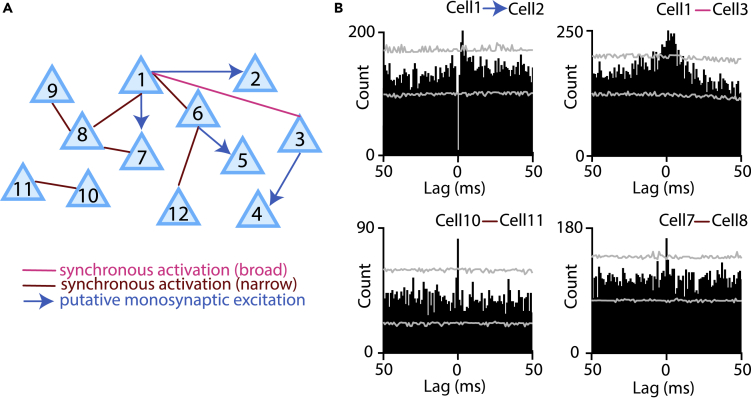
VP neurons form co-firing assemblies (A) An example cell assembly formed by VP neurons during Pavlovian conditioning. Brown, narrow (1–2 ms) zero-lag synchrony; pink, broad (≥3 ms) zero-lag synchrony; blue, putative monosynaptic excitation. (B) Example cross-correlograms of neuronal pairs of this assembly. Gray, 95% confidence intervals.

Neurons in some cortical and subcortical areas have been shown to form functional assemblies of co-firing cells (Dupret et al., 2010; Fujisawa et al., 2008). We performed a cross-correlation analysis of simultaneously recorded pairs of VP neurons (n = 4,942) and found many indications of functional connectivity. Neuronal pairs often showed a zero-lag peak of cross-correlation typically taken as an indication of a common input. Narrow (1–2 ms wide) peaks within 1–4 ms from 0, on the other hand, usually indicate monosynaptic excitatory connections (Bartho et al., 2004; Fujisawa et al., 2008). We could identify small networks of VP neurons exhibiting pairwise synchrony, suggestive of assembly formation during associative learning in the VP (Figure 7).

### Neurons participating in assemblies respond more frequently to reinforcement

**Figure 8:**
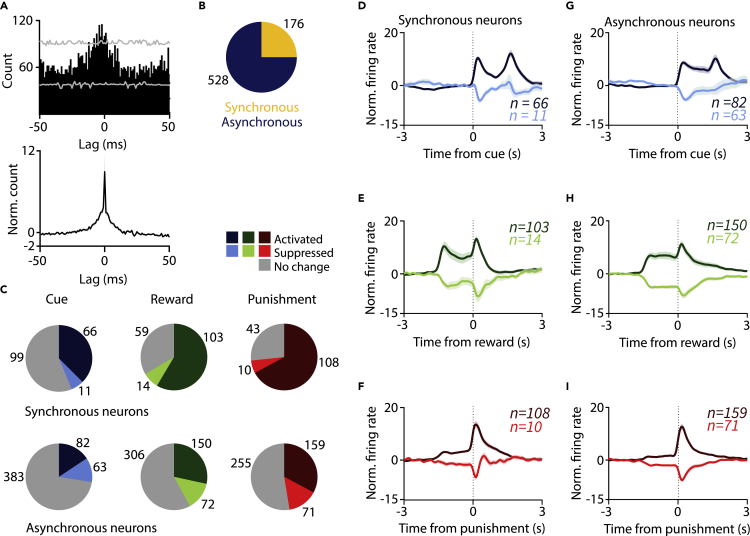
Synchronous neurons respond more to reinforcement (A) Top, example cross-correlogram of synchronously activated neurons. Bottom, average cross-correlation of all synchronously active pairs. (B) Pie chart showing the proportion of synchronous and asynchronous VP neurons, pooled across mice. (C) Pie charts showing the number of synchronous and asynchronous VP neurons activated or inhibited by cue, reward or punishment, pooled across mice. (D–I) Average, *Z*-scored PETHs of synchronous (D–F) and asynchronous (G–I) VP neurons aligned to cue (D and G), reward (E and H), and punishment (F and I). Data are represented as mean ± SEM. See also Figure S9.

VP neurons were sorted based on their cross-correlograms. Neurons that participated in synchronously firing assemblies based on significant zero-phase peaks detected in pairwise cross-correlations (Figure 7) were termed “synchronous neurons,” whereas neurons wherein no such concurrent activation was observed were called “asynchronous neurons” (Figures 8A and 8B). Although this distinction probably mislabels some neurons that participate in assembly formation as “asynchronous” due to missed detections, we still uncovered prominent differences between the two groups. Neurons that participated in the detected assemblies (“synchronous group”) showed higher baseline firing rates (Figure S6C) and more frequent responses to cue, reward, and punishment compared with those neurons for which we did not detect synchronous pairs (“asynchronous group”; p = 5.64 × 10^−5^, p = 1.93 × 10^−8^, p = 6.79 × 10^−8^ for cue, reward, and punishment response, respectively; chi-square test; Figures 8C–8I and S9). These differences probably represent an underestimation, because it is likely that we missed a fraction of synchronous activations.

Our results on bursting versus non-bursting, rhythmic versus non-rhythmic, and synchronous versus asynchronous neurons were not altered when neurons that potentially overlapped across recording sessions performed with small differences in dorsoventral position were excluded (n = 42/704; Figure S10; see transparent methods).

### Topography of electrophysiological properties within the VP

**Figure 9:**
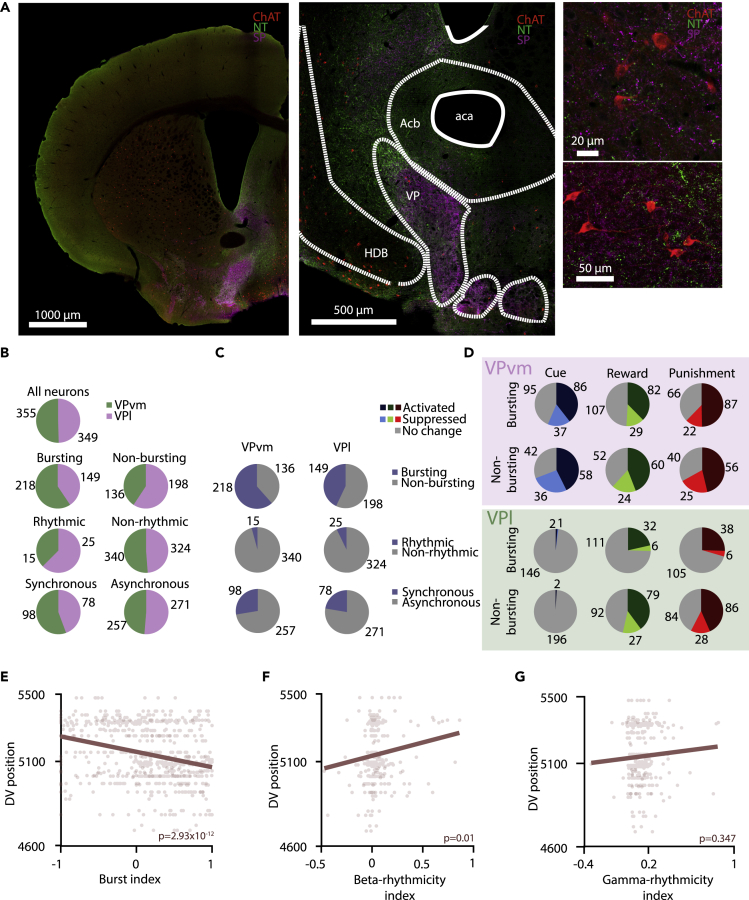
Comparison of ventromedial (VPvm) and lateral (VPl) subregions (A) Fluorescent images of anti-ChAT (red), anti-NT (green), and anti-SP (magenta) triple immunohistochemical staining. Left, coronal section of a hemisphere. Scale bar, 1,000 μm. Middle, VP area. Scale bar, 500 μm. Right, high-magnification images of VP cholinergic neurons. Scale bars, 20 and 50 μm. (B) Pie charts showing the proportion of VPvm versus VPl neurons within all recorded VP neurons and broken down to the different e-types examined. (C) Pie charts showing the proportion of bursting versus non-bursting, rhythmic versus non-rhythmic, and synchronous versus asynchronous neurons across the VPvm and VPl subpopulations. (D) Pie charts showing the proportion of neurons activated or suppressed after presentation of the cue, the reward, or the punishment. Bursting versus non-bursting neurons are shown for VPvm and VPl separately. (E–G) Correlation of the burst index (E), beta-rhythmicity index (F), and gamma-rhythmicity index (G) with dorsoventral position of recorded VP neurons. Burst index showed significant negative (p < 0.001) and beta-rhythmicity index showed significant positive correlation (p = 0.01) with dorsoventral position.

The VP is not a homogeneous anatomical structure: based on afferent and efferent innervation patterns and marker expression, ventromedial (VPvm), dorsolateral (VPdl), ventrolateral (VPvl), and rostral (VPr) subnuclei have been described (Root et al., 2015). To differentiate ventromedial and lateral parts of the VP, we carried out triple immunostainings of choline-acetyltransferase (ChAT), neurotensin (NT), and substance P in naive mice. The area containing ChAT+ neurons and SP+ fibers marked the VP, within which the VPvm was differentiated by NT+ fibers (Figure 9A and Table 1). The immunostainings and the recording positions of electrophysiologically characterized VP neurons were aligned to a common atlas reference (Franklin and Paxinos, 2007) (see transparent methods, histology), allowing us to determine whether VP neurons were recorded from the VPvm versus lateral parts of the VP, referred to VPl hereafter. We found that the VPvm was characterized by a significantly larger fraction of bursting neurons than the VPl (p = 7.77 × 10^−7^, chi-square test), whereas no significant differences in the ratio of rhythmic versus non-rhythmic and synchronous versus asynchronous neurons was detected (Figures 9B and 9C). Our observation that non-bursting, non-rhythmic VP neurons were more responsive to salient stimuli remained consistent across VPvm and VPl (Figure 9D). Finally, we discovered a dorsoventral shift in electrophysiological properties, where burst index showed a significant negative (p = 2.93 × 10^−12^) and beta rhythmicity index showed a significantly positive correlation (p = 0.01) with dorsoventral position, suggesting that dorsal VP neurons fire more bursts and tend to be less rhythmic compared with ventral VP (Figures 9E–9G). As most of the VPl is located ventrally to VPvm (Figure 9A), this finding is consistent with the larger proportion of burst firing neurons in VPvm.

**Table 1:** Antibodies used for immunohistochemistry

Raised against	Host	Vendor	Catalog no.	Concentration
Choline acetyltransferase	Goat	Millipore	AB144P-200UL	1:500
Neurotensin	Guinea pig	Synaptic Systems	418 005	1:500
Substance P	Rabbit	Immunostar	20064	1:1000

## Discussion

The role of VP in reward-related and motivated behavior has been extensively studied; however, there have been very few attempts to distinguish electrophysiologically defined neuronal populations, i.e., e-types (Gouwens et al., 2019), during reinforcement learning (Avila and Lin, 2014; Kaplan et al., 2020). Therefore, the aim of this study was to characterize electrophysiologically distinct functional groups within the VP during reinforcement learning. We found that most responses to reward and punishment in the VP originated from a group of non-bursting-non-rhythmic neurons, suggesting that this population is dominant in representing reinforcers in the VP. Importantly, a subpopulation of bursting neurons showed differential responses when their bursts were contrasted with their single spikes, demonstrating that a specific “burst code” may be present in the VP (Kepecs and Lisman, 2003; Laszlovszky et al., 2020). VP neurons formed co-firing assemblies, and neurons participating in such assemblies were particularly responsive to reinforcement. We propose that electrophysiologically defined e-types of the VP differentially participate in transmitting reinforcement signals during learning.

Many studies have discussed the role of the VP in learning cue-reward associations (Ahrens et al., 2018; Avila and Lin, 2014; Fujimoto et al., 2019; Ito and Doya, 2009; Ottenheimer et al., 2018; Richard et al., 2016, 2018; Tachibana and Hikosaka, 2012; Tindell, 2004) as well as in adapting behavioral responses to outcome during reinforcement learning (e.g., “liking” reactions after reward delivery and “disgust” reactions to aversive stimuli) (Ho and Berridge, 2014; Smith and Berridge, 2005; Tindell et al., 2006), gated by internal state (Chang et al., 2017; Stephenson-Jones et al., 2020). Many of these experiments only included rewarded and omitted trials, whereas comparatively fewer articles featured aversive stimuli (Kaplan et al., 2020; Knowland et al., 2017; Saga et al., 2017; Stephenson-Jones et al., 2020; Wulff et al., 2019). Cued reward size modifications were often included (Stephenson-Jones et al., 2020; Tachibana and Hikosaka, 2012), although how the VP adapts to probabilistic cues that are notoriously harder to learn, because they require integration over many trials, has remained largely unexplored.

Therefore, we trained mice on a Pavlovian reinforcement learning task where we incorporated both reward and punishment into our task design, in order to examine VP neuronal activity patterns upon both positive and negative outcomes. We found a VP population activated by both reward and reward-predicting cues, consistent with previous findings (Ahrens et al., 2016, 2018; Stephenson-Jones et al., 2020; Tindell, 2004). However, we also found a comparable number of punishment-responsive VP neurons that have largely been overlooked before. Moreover, responses to punishment were significantly faster than reward or cue-elicited firing rate changes.

A significant population showed inhibition to cues and reinforcement regardless of valence, consistent with Type III GABAergic neurons in the study by Stephenson-Jones et al. (2020). However, we found very few neurons that responded with opposite firing rate changes to reward and punishment, unlike in the above-cited report, and in most cases the signs of responses were correlated across stimuli (Figure 2O). An important factor that likely underlies these differences is that we used a probabilistic task design, in which both cues were followed by reward, punishment, or nothing with different, set probabilities. Thus, probabilistic expectations provide a task context in which VP neurons tend to respond more positively, as all cues carry some positive value, also indicated by the dominance of neuronal activation versus inhibition in our recordings. Additionally, a number of these cells might be modulated by incentive salience rather than encoding outcome valence, as found in previous studies (Ahrens et al., 2018; Stephenson-Jones et al., 2020; Tindell, 2004; Tindell et al., 2009). We also note that most of our recordings originated from the anterior half of the VP, thus known anatomical differences along the anteroposterior axis (Mahler et al., 2014; Stratford et al., 1999) may have contributed to some of these differences.

The VP is a key node in the integration of limbic and motor processes (Fujimoto et al., 2019). The probabilistic outcome contingencies of our Pavlovian task enabled us to show that VP neurons' cue responses are modulated by reward expectation. Moreover, both the abundance of cue responses and the depth of modulation by expectation correlated with behavioral discrimination of the probabilistic cues (Stephenson-Jones et al., 2020). This finding is consistent with previous findings showing populations of VP neurons represent incentive salience (Ahrens et al., 2016, 2018; Tindell et al., 2005) or outcome prediction errors (Kaplan et al., 2020; Ottenheimer et al., 2020). The VP is known to be strongly innervated by dopaminergic fibers arising from the VTA. It appears that the functional role of this connection is the modulation of locomotion by acting on VP neuronal output (Klitenick et al., 1992). Moreover, the VTA itself is also considered to be crucial for reward expectation coding (Hollerman and Schultz, 1998) and has been shown to promote place preference via its afferent projections from the VP (Faget et al., 2018). The strong reciprocal connection between the two areas could serve as a neural basis of reward-seeking behavior in rodents. We should note, however, that the VP also lies at the intersection of basal ganglia and basal forebrain circuits, the latter also featuring prominent reward prediction activity and salience coding (Avila and Lin, 2014; Hangya et al., 2015; Lin and Nicolelis, 2008); therefore, multiple origins of such signals are feasible. In this regard, an important finding showed that VP responses to reward appear earlier than those in the nucleus accumbens, making the previously hypothesized accumbens to VP information transfer less likely (Ottenheimer et al., 2018; Richard et al., 2016).

We characterized the activity of bursting VP neurons and found that they were less responsive to reward and punishment than non-bursting neurons. This was surprising, as bursts of action potentials are often thought to be associated with stronger excitatory drive that may lead to larger firing rate increases. Indeed, in the basal forebrain, bursting neurons as well as burst responses were associated with populations responsive to salient stimuli (Laszlovszky et al., 2020; Lin and Nicolelis, 2008). Multiple biophysical mechanisms can generate bursts of action potentials (Kim et al., 2015a; Otomo et al., 2020; Yang et al., 2018) with different temporal dynamics. We tested whether this finding was dependent on our exact burst definitions but found higher responsiveness of non-bursting neurons even when slower bursts up to 30 ms ISIs were included.

Different higher order firing patterns may represent specific information, as a special case of temporal code (Panzeri et al., 2010). Thus, bursts of action potentials conceivably code different variables from single spikes even within single neurons, allowing within-cell multiplexing of information (Kepecs and Lisman, 2003; Kepecs et al., 2002). For instance, bursts of visual thalamic neurons were shown to have sharper tuning than single spikes (Reinagel et al., 1999), and basal forebrain bursts of both cholinergic and non-cholinergic neurons represent specific information about salient stimuli (Hangya et al., 2015; Laszlovszky et al., 2020; Lin and Nicolelis, 2008). In accordance, we found VP neurons that showed strong differences in their burst and single spike occurrence after salient stimuli, which could in some cases change in opposite directions (Figure 6).

Pang and colleagues identified a fast rhythmic type of VP neuron, with rhythmicity frequency in the beta/gamma bands (Figure 8A in Pang et al., 1998). Recently, VP gamma activity was linked to somatostatin (SOM)-expressing GABAergic neurons that influenced movement speed (Espinosa et al., 2019). In contrast, SOM neurons in the medial septum did not exhibit gamma correlation. Indeed, in other parts of the basal forebrain, gamma oscillations were better correlated with parvalbumin-expressing GABAergic neurons (Kim et al., 2015b). This basal forebrain-VP dissociation posits that these SOM GABAergic VP neurons may be more linked to basal ganglia than basal forebrain activity. Consistent with this, we found that these fast-rhythmic neurons are not prominent contributors of VP reinforcement responses.

The presence of cell assemblies has previously been demonstrated in the hippocampus, nucleus accumbens, and basal forebrain (Harris et al., 2003). Dynamically forming cell assemblies of the hippocampus (Harris et al., 2003; Tingley et al., 2015; Trouche et al., 2019) were linked to spatial navigation and episodic memory recall (Dupret et al., 2010; Mamad et al., 2017; Pastalkova et al., 2008). Assemblies in the basal forebrain were suggested to organize behavior in an attention task (Tingley et al., 2014, 2015). We demonstrated that synchronously firing cell assemblies are also formed in the VP during Pavlovian conditioning. Moreover, the temporal scale of co-firing closely matched the 10–30 ms previously suggested to be a conserved parameter under biophysical constraints (Harris et al., 2003) (Figure 8A). Consistent with the idea that “transient synchrony of anatomically distributed groups of neurons underlies processing of both external sensory input and internal cognitive mechanisms” (Harris et al., 2003), we found that neurons participating in co-firing ensembles were more responsive to behaviorally important sensory signals during Pavlovian learning.

VPvm receives projections from nucleus accumbens shell and VTA neurons (Root et al., 2015). It was proposed to participate in discriminating rewarding signals, serve consummatory behavior, and participate in working memory (Jenck et al., 1986; Kalivas et al., 2001; Root et al., 2015). In contrast, VPdl receives nucleus accumbens core input and is thought to control motor output through its projections to the subthalamic nucleus (STN) and substantia nigra pars reticulata (SNr). Little is known about behavioral functions of VPvl. However, VPvm activity has also been linked to motor functions and aversion (Kitamura et al., 2001). Given that VPvm and VPdl participate in overlapping functions related to reward and punishment, it is not surprising that both VPvm and VPl neurons responded to reinforcement in the probabilistic Pavlovian task. The nucleus accumbens core-VPdl-STN/SNr route is thought to serve as an indirect motor control pathway, relevant for movement initiation, execution, and stopping (Root et al., 2015; Tripathi et al., 2010, 2013). This strong involvement in movement control may explain the higher proportion of non-bursting neurons within the VPl, generally more sensitive to salient stimuli. Nevertheless, VP subregions are embedded in partially overlapping anatomical circuits, and better understanding of their functional distinctions will require further experiments.

The output of VP neurons can be modulated by enkephalins and dopamine, because VP neurons express μ- and κ-opioid receptors as well as D1 and D2 receptors (Clark and Bracci, 2018; Kupchik et al., 2015; Panagis et al., 1998). This receptor pattern makes the VP especially sensitive to drugs targeting the dopaminergic and opioid system, including opiates and cocaine (Creed et al., 2016; Heinsbroek et al., 2020; Mahler et al., 2014; Mickiewicz et al., 2009). A shift in the activity pattern of VP neurons can lead to maladaptive behavior, such as perseveration or intracranial self-stimulation in rodents, or severe addiction in humans (Hubner and Koob, 1990; Ottenheimer et al., 2019). Moreover, in patients with long-term addiction, the pathological function is accompanied by morphological changes (Müller et al., 2019). Therefore, the VP might be an ideal target for the treatment of drug addiction and habitual relapse. The nucleus accumbens, one of the main inputs to the VP, has already been proposed as a potential target for deep brain stimulation (DBS) in patients with addiction (Kuhn et al., 2014; Müller et al., 2015). However, the central position of the VP in the reward circuitry and its strong relation to addiction could make the VP another plausible target for DBS (Mahoney et al., 2018; Yu et al., 2016). Gaining a better foothold on understanding the activity patterns and coding schemes of the VP will be fundamental for developing an effective stimulation protocol while minimizing side effects.

### Limitations of the study

In this study, we provide a comprehensive analysis of VP electrophysiologically defined cell types along several dimensions. We used a behavioral task that features the learning of probabilistic cue contingencies and both aversive and appetitive stimuli. However, the study has a number of limitations that should be addressed in future experiments. First, the potential nonspecific influence of genetic background, type of knock-in transgene, and viral injections has not been evaluated. Future studies comparing wild-type mice of different genetic background may shed light on behavioral differences across mouse strains with relevance to VP function. Second, the VP contains behaviorally relevant subdivisions of lateral VP and a small rostral nucleus (VPr), which we were unable to reliably discriminate with tetrode recordings. Optogenetic tagging of specific projection neurons by retrogradely spreading viral vectors could resolve this in the future. Third, we targeted the anterior part of the VP; therefore, potential differences between anterior and posterior locations could not be addressed. Fourth, although we attempted to discount for potential overlap in recorded neurons across recording days (see transparent methods), this lowers the sample size of independent neurons. Further subdivision of VP neurons based on either anatomical parcellation or more electrophysiological properties would require larger samples of VP neurons recorded in behaving mice.

### Resource availability

#### Lead contact

Further information and requests for resources should be directed to and will be fulfilled by the lead contact, Balázs Hangya (hangya.balazs@koki.hu).

#### Materials availability

This study did not generate new unique reagents.

#### Data and code availability

MATLAB code developed to analyze the data presented in this study is available at www.github.com/hangyabalazs/VP_data_analysis. Electrophysiology and behavioral data are available from the lead contact upon reasonable request.

## Methods

All methods can be found in the accompanying Transparent Methods supplemental file.

## Supplemental information

Document S1. Transparent methods and figures S1–S10
