# Supplementary material for: Differential recruitment of ventral pallidal e-types by behaviorally salient stimuli during Pavlovian conditioning
Source: iScience. 2021 Mar 31;24(4):102377. doi: 10.1016/j.isci.2021.102377 (PMC8066429; doi:10.1016/j.isci.2021.102377)
Supplement: Document S1. Transparent methods and figures S1–S10 [file mmc1.pdf]

iScience, Volume *24*

Supplemental information

Differential recruitment of ventral pallidal e-types by behaviorally salient stimuli during Pavlovian conditioning

Panna Hegedüs, Julia Heckenast, and Balázs Hangya

Supplemental Figures

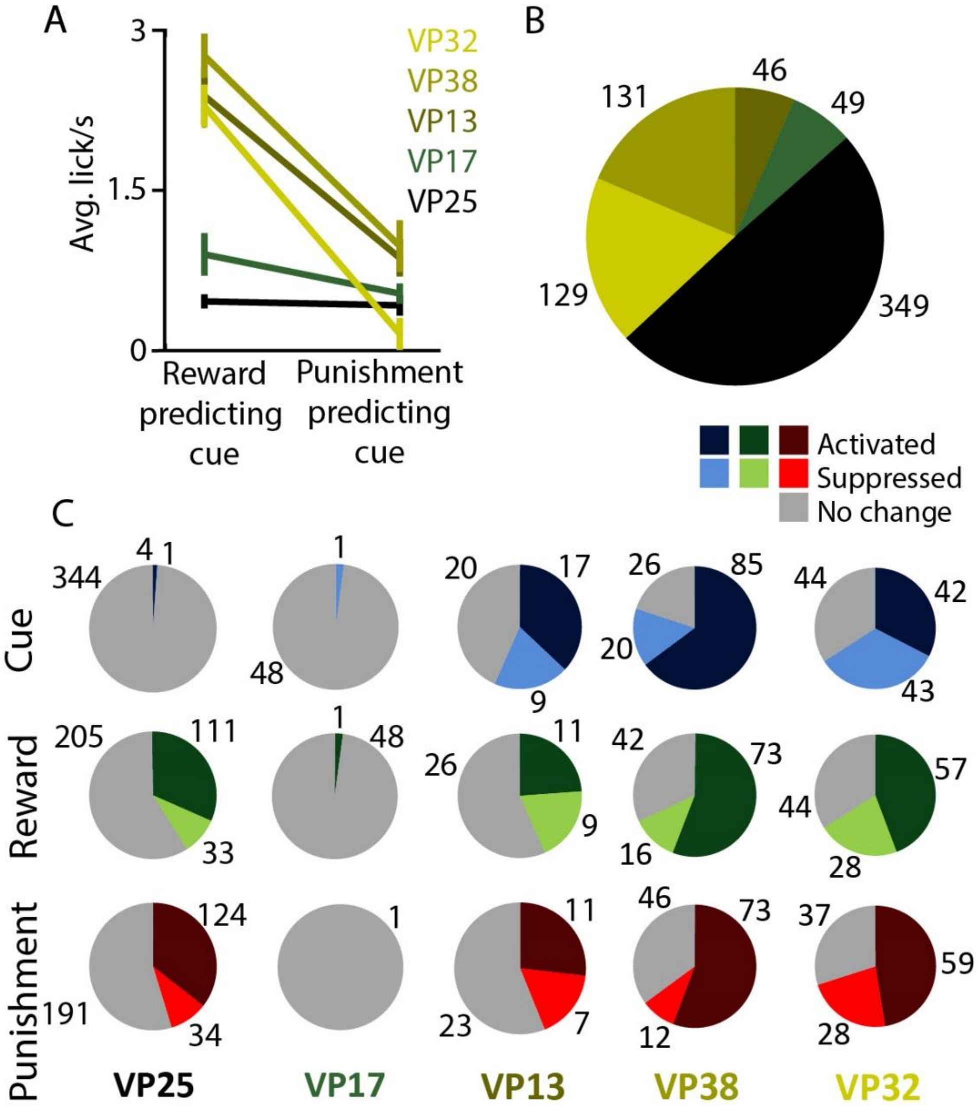

Figure S1 (Related to Figure 2). The number of cue and reinforcement responsive cells correlates with behavior. ***A,*** Line plot of anticipatory lick rate difference for reward and punishment predicting cue. Lines, corresponding to individual mice, are color coded based on anticipatory lick rate difference (yellow – large anticipatory lick rate difference, black – small anticipatory lick rate difference). Data are represented as median ± SE of median. ***B,*** Pie chart showing the number of neurons recorded in each animal (N = 5 mice). ***C,*** Pie charts showing the number of cue, reward and punishment responsive neurons in each animal.

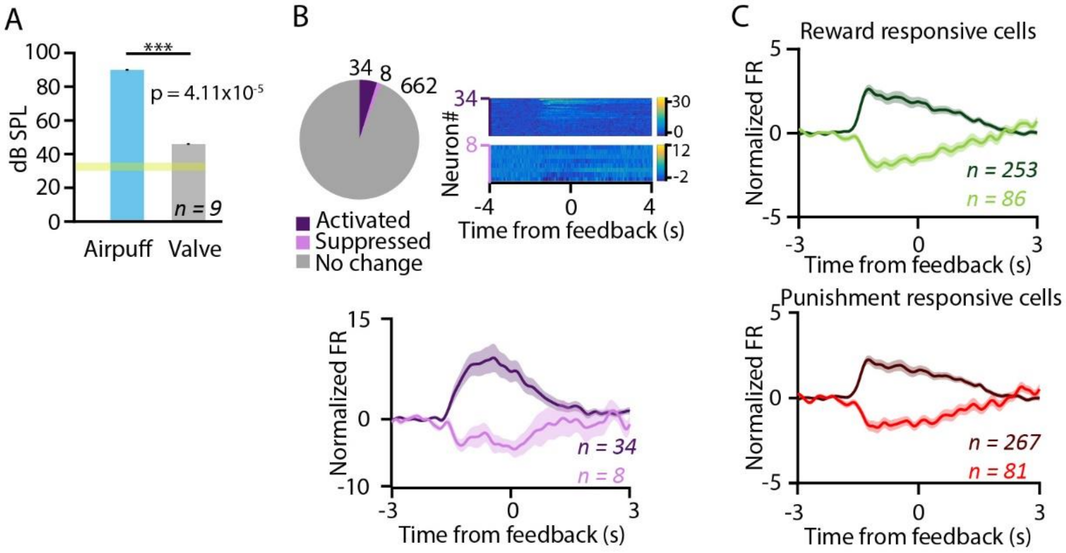

Figure S2 (Related to Figure 2). Responses to outcome omissions were rare in the VP. ***A,*** We measured the sound pressure level associated with the flow of air at air-puff punishment, the sound pressure level of the click sound of the solenoid valve at reward presentation and the ambient background noise (yellow shading). ***, p < 0.001, Mann-Whitney U-test. ***B,*** Pie chart showing the number of neurons that changed their firing rates significantly compared to baseline after the time point of expected but omitted reinforcement. Only few neurons showed significant firing rate differences after the time point of omitted reinforcement. These responses appeared to be better explained by the cue presentations. Data are represented as mean ± SEM. ***C,*** Peri-event time histograms of VP neurons responsive to reward or punishment, aligned to the time point of omitted reinforcers. Reward- and punishment-responsive neurons showed a smooth decay of cue-related responses at the time of omitted feedback. Data are represented as mean ± SEM.

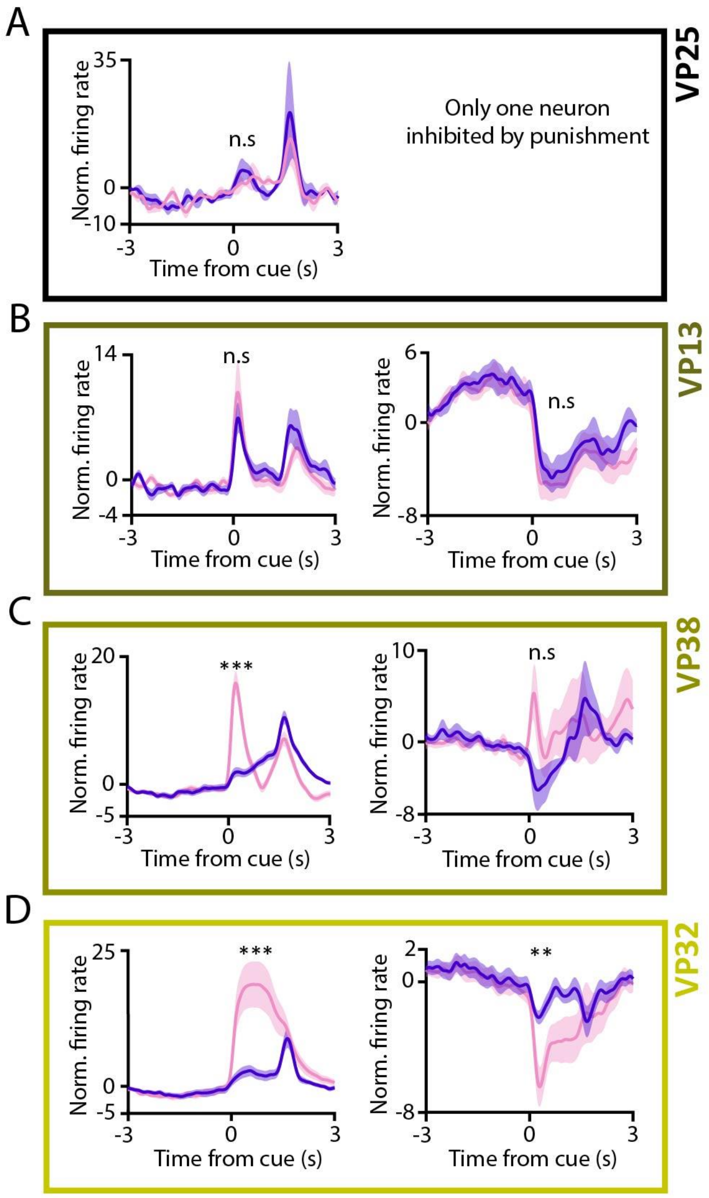

Figure S3 (Related to Figure 3). Differential neuronal response to reward and punishment predicting cues correlates with anticipatory lick difference. Average PETH of VP neuronal activation in animal VP25 ***(A)***, VP13 ***(B)***, VP38 ***(C)*** and VP32 ***(D)*** after cues predicting likely reward (pink) or likely punishment (purple). Color code corresponds to anticipatory lick rate difference (yellow – large anticipatory lick rate difference, black – small anticipatory lick rate difference). Data are represented as mean ± SEM. **, $p < 0.01$; ***, $p < 0.001$, Wilcoxon signed rank test.

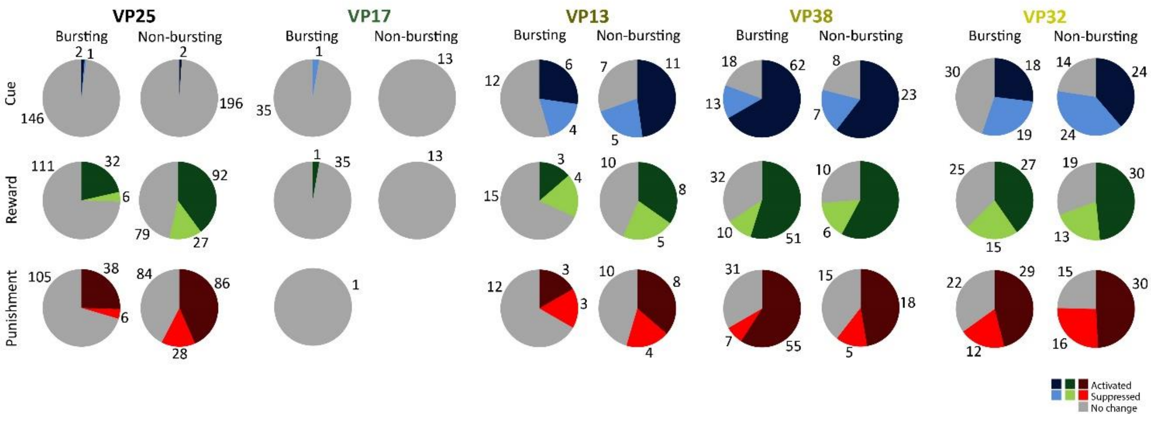

Figure S4 (Related to Figure 4). Pie charts showing the number of bursting and non-bursting cells modulated by cue, reward or punishment for individual mice. Color code of the animal corresponds to anticipatory lick rate difference (yellow – large anticipatory lick rate difference, black – small anticipatory lick rate difference).

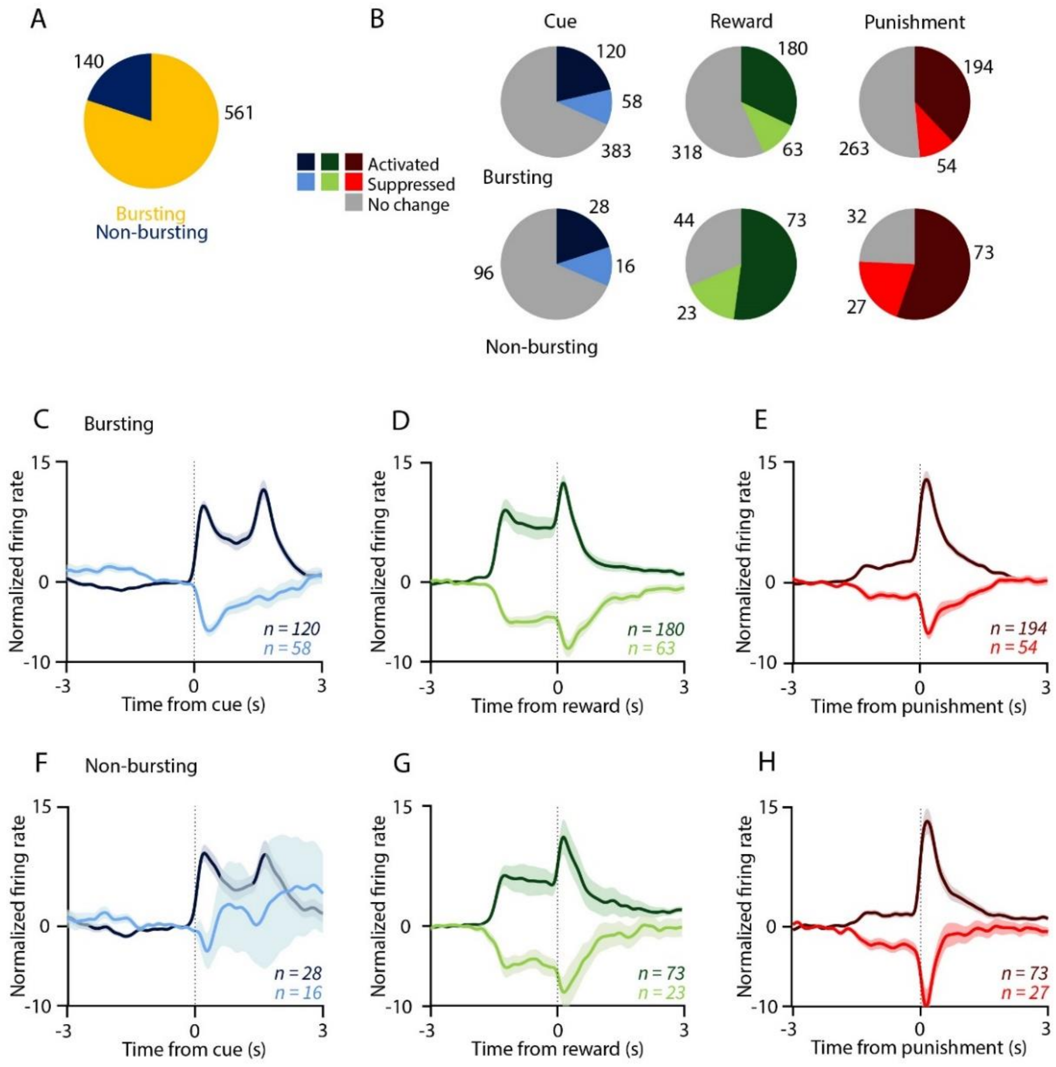

Figure S5 (Related to Figure 4). Non-bursting VP neurons respond to reinforcers more frequently, independent of ISI cutoff. ***A,*** Pie chart showing the proportion of bursting and non-bursting neurons when bursts were defined by using a 30 ms ISI cut-off. ***B,*** Pie charts showing the number of bursting and non-bursting VP neurons activated or inhibited by cue, reward or punishment. **C-H,** Average, z-scored PETHs of bursting ***(C-E)*** and non-bursting ***(F-H)*** VP neurons aligned to cue ***(C,F)*** reward ***(D,G)*** and punishment ***(E,H)***. Data are represented as mean ± SEM.

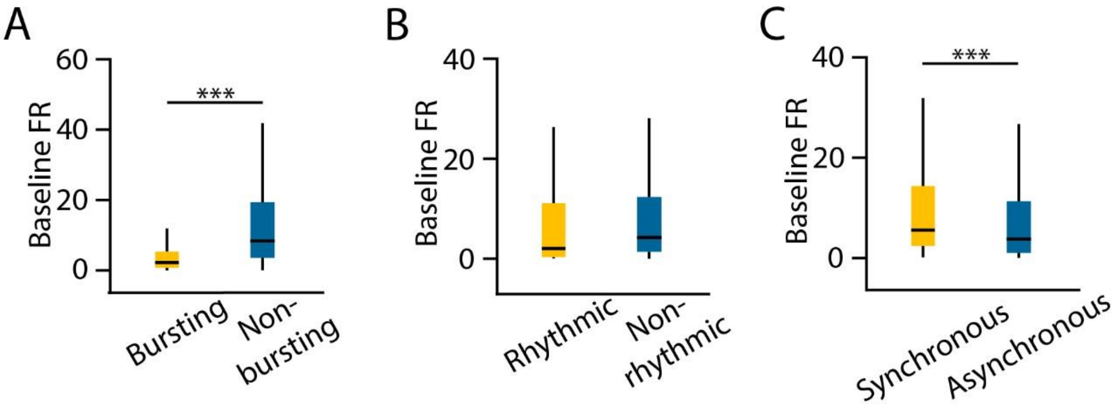

Figure S6 (Related to Figure 4). Firing rate distributions of VP e-types. ***A,*** Baseline firing rate of bursting and non-bursting neurons. ***B,*** Baseline firing rate of rhythmic and non-rhythmic neurons. ***C,*** Baseline firing rate of synchronous and asynchronous neurons. Box-whisker plots represent median, interquartile range and non-outlier range. ***, $p < 0.001$, Mann-Whitney U-test

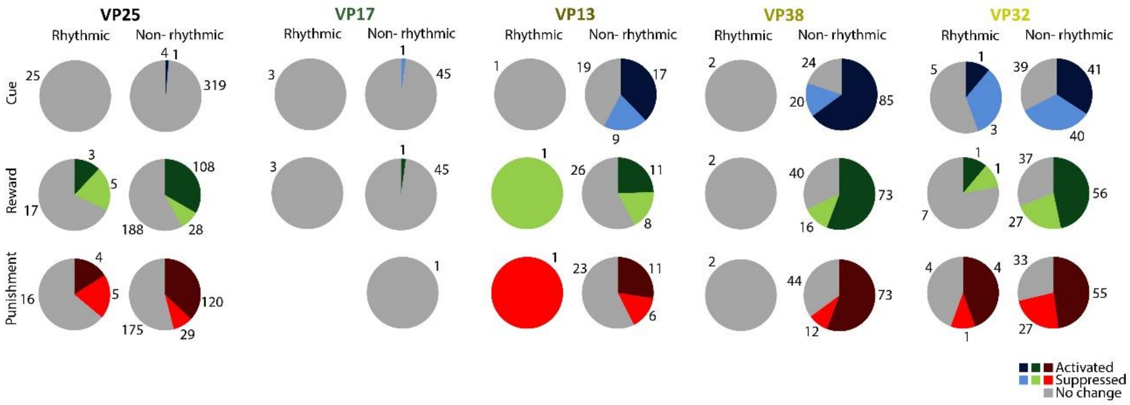

Figure S7 (Related to Figure 5). Pie charts showing the number of rhythmic and non-rhythmic neurons modulated by cue, reward or punishment for individual mice. Color code of the animal corresponds to anticipatory lick rate difference (yellow – large anticipatory lick rate difference, black – small anticipatory lick rate difference).

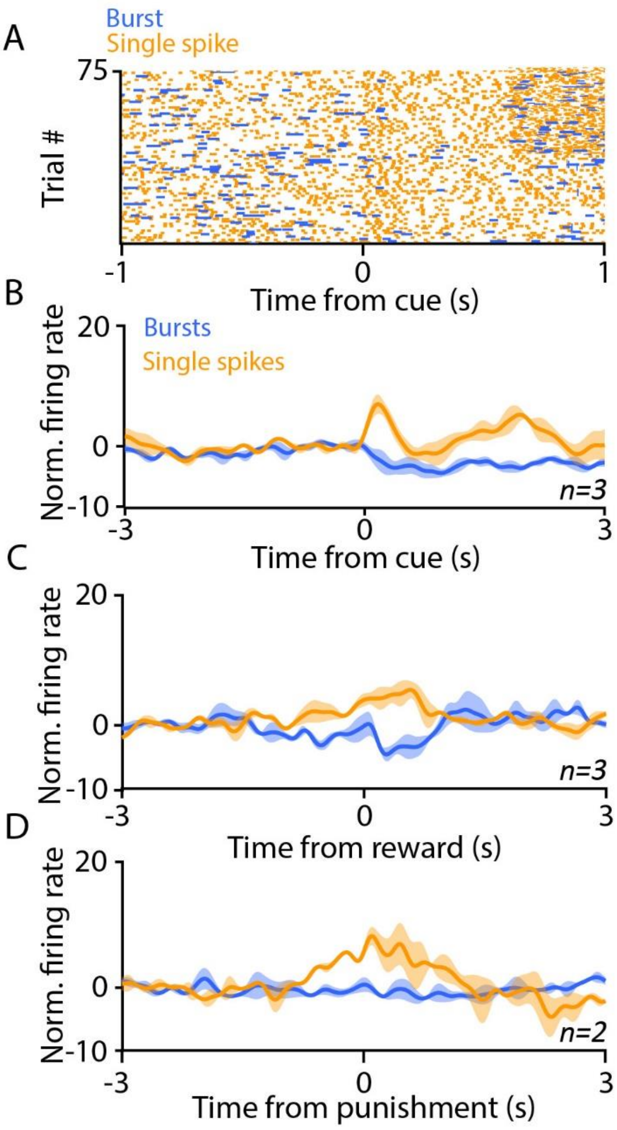

Figure S8 (Related to Figure 6). Dissociation of burst and single spike responses. ***A,*** Bursts (blue ticks) and single spikes (orange ticks) of an example neuron. Note that burst firing is decreased meanwhile single spike firing is increased upon cue presentation. ***B-D,*** Average z-scored PETH of neurons with increased single spike (orange) and decreased burst (blue) activity aligned to behaviorally salient events. Data are represented as mean ± SEM.

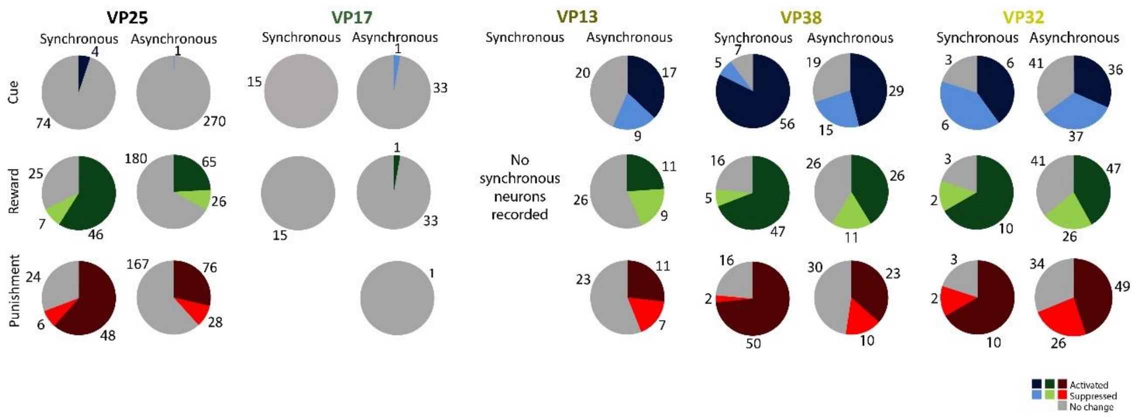

Figure S9 (Related to Figure 8). Pie charts showing the proportion of synchronous and asynchronous neurons modulated by cue, reward or punishment for individual mice. Color code of the animal corresponds to anticipatory lick rate difference (yellow – large anticipatory lick rate difference, black – small anticipatory lick rate difference).

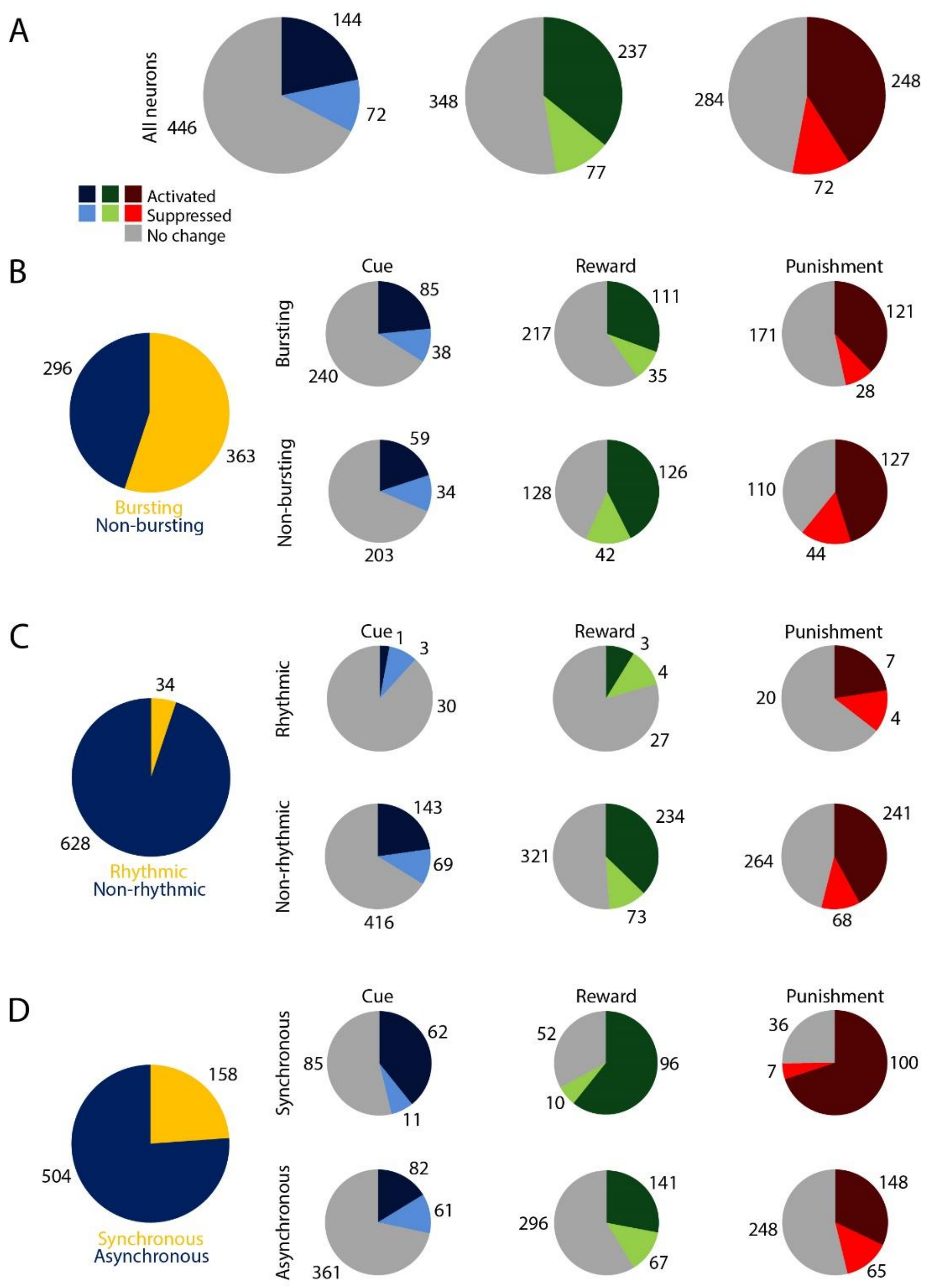

Figure S10 (Related to Figure 8). Proportion of VP e-types after potential duplicates of recorded neurons were excluded. ***A,*** Pie charts of cue, reward and punishment responsive VP cells. **B,** Left, pie chart showing the proportion of bursting and non-bursting cells. Right, pie charts showing the proportion of bursting and non-bursting VP neurons activated or inhibited by cue, reward or punishment. ***C,*** Left, pie chart showing the proportion of rhythmic and non-rhythmic cells. Right, pie charts showing the proportion of rhythmic and non- rhythmic VP neurons activated or inhibited by

cue, reward or punishment. ***D,*** Left, pie chart showing the proportion of synchronous and asynchronous cells. Right, pie charts showing the proportion of synchronous and asynchronous VP neurons activated or inhibited by cue, reward or punishment.

Transparent Methods

Animals

Adult male mice (n = 4 ChAT-IRES-Cre, B6129F1 and n = 1 PV-IRES-Cre, FVB/AntFx, VP17) were used for recording and C57Bl/6J male mice (n = 2) were used for immunohistochemistry according to the regulations of the European Community's Council Directive of November 24, 1986 (86/609/EEC). Experimental procedures were reviewed and approved by the Animal Welfare Committee of the Institute of Experimental Medicine, Budapest and by the Committee for Scientific Ethics of Animal Research of the National Food Chain Safety Office of Hungary.

Surgery

Mice were anesthetized with an intraperitoneal injection of ketamine-xylazine (0.166 and 0.006 mg/kg, respectively) after a brief induction with isoflurane. After shaving and disinfecting the scalp (Betadine), the skin was infiltrated with Lidocaine and the eyes were protected with eye ointment. Mice were placed in a stereotaxic frame and the skull was levelled along both the lateral and the antero-posterior axes. The skin, connective tissues and periosteum were removed from the skull and a cranial window was drilled above the anterior ventral pallidum (antero-posterior 0.75 mm, lateral 0.6 mm). Two additional holes were drilled above the parietal cortex for ground and reference. After virus injection to the VP (AAV 2/5. EF1a.Dio.hChR2(H134R)-eYFP.WPRE.hGH), a custom-built microdrive (Hangya et al., 2015; Kvitsiani et al., 2013) was implanted into the VP using a cannula holder on the stereotactic arm. The choice of the transgenic mouse lines and the viral construct was motivated by the prospect of optogenetic tagging (not reported). Specific expression of the fluorophore helped verifying the reconstruction of the tetrode tracks (see below). The microdrive and

a titanium headbar were secured to the skull with dental cement (LangDental acrylic powder and liquid resin, C&B Metabond quick adhesive cement). The analgesic buprenorphine (Bupaq) was administered, and mice were allowed a 1-week recovery period and handled for an additional week before training and recording.

Pavlovian cued outcome task protocol

Mice were trained on an auditory Pavlovian conditioning task in a head-fixed behavioral setup described in detail previously (Solari et al., 2018). On the first day of training, thirsty mice were head-fixed and given free access to water reward whenever they licked a waterspout. The next day, a pure tone cue was introduced that predicted likely reward. After each cue presentation, water reward was delivered with 0.8 probability with a 400-600 ms delay, while the rest of the outcomes were omissions. Next, a second pure tone cue of well-separated pitch was introduced that predicted reward with low probability (0.25). Air puff punishment (200 ms, 30 psi) was introduced in the following session with the final outcome contingencies (likely reward trials, 80% reward, 10% punishment, 10% omission; likely punishment trials, 25% reward, 65% punishment, 10% omission). The trials with different trial types (likely reward and likely punishment) and outcomes (water reward, air puff punishment and omission) were presented in a pseudorandomized order. Mice learned the task in approximately one week and consistently demonstrated reward anticipation by differential lick rate in response to the cues from the second week (Figures 1E-H).

Sound output of air-puffs and low-noise solenoid valves (LHDA0531115H, The Lee Company) were measured using a calibrated precision electret condenser microphone (EMM-6, Daytonaudio) connected to a preamplifier digital converter (AudioBox iOne, PreSonus); sound pressure levels were measured by the TrueRTA software (see (Solari et al., 2018) for more details on sounds calibration methods).

Recording

Extracellular recordings were performed with custom made microdrives consisting of 8 movable tetrode electrodes and an optical fiber. Microdirive screws were specifically designed and machined, optimized for small size and weight and having an unusually small pitch of the threading (160 µm) to allow precisely controlled descent in the brain (one eighths of a turn corresponded to 20 µm descent; M0.6 stainless steel flat head screw, 12 mm length; Easterntec, Shanghai, China). We measured the protruding length of the electrodes on each microdrive before surgery (Olympus SZ61 stereomicroscope; micro-ruler, Electron Microscopy Tools). The electrodes were dipped in DiI red fluorescent dye to aid later track reconstruction efforts.

Before each recording session, the microdrive was connected (Omnetics) to a 32-channel RHD headstage (Intan). Data were digitized at 30 kHz and transferred from the headstage to a data acquisition board (Open Ephys) via a Serial Peripheral Interface cable (Intan). The tetrodes were advanced 0-100 µm after each recording session. Throughout the experiments, detailed notes of the assumed brain coordinates during each recording session were taken based on the measured length of the tetrodes, stereotaxic information from the surgery and controlled screw turns on the microdrive.

Histology

After the *in vivo* experiments, animals were anesthetized with an intraperitoneal injection of ketamine-xylazine (0.166 and 0.006 mg/kg, respectively) and underwent an electrolytic lesioning protocol (30 µA for 5s on two leads of two selected tetrodes, which had provided high yield of recorded units; stimulator from Supertech, Pecs, Hungary). Mice were then and transcardially perfused with saline for 2 minutes and 4% para-formaldehyde (PFA) for 20 minutes. The brain was gently removed from the skull, postfixed in PFA overnight and then washed in phosphate buffer. The explanted microdrives were examined under stereomicroscope and the protruding length of the electrodes were verified against the depth registrations of the Experimenter. Coronal sections of 50 µm thickness were cut by a vibratome (Leica VT1200S). Special care was taken to section the brain

perpendicular to brain surface, so that resulting sections were in plane with coronal atlas images. The sections were washed in phosphate buffer 3 times and mounted on microscopy slides in Aquamount mounting resin. Fluorescent micrographs of the sections were taken using a Nikon C2 confocal microscope. We took 4x4 large field-of-view dark-field, red and green fluorescent images at 10x magnification.

The images taken by the confocal microscope were further processed to recover the recording location of each recording session referenced to atlas coordinates (Paxinos et al., 2001). These adjustments could account for individual size differences of mouse brains compared to the atlas reference and slight deviations from the vertical direction during electrode descent. Dark-field whole-section brain images were used for atlas alignment, since they provided the best contrast for white and grey matter structure of the brain. Atlas images of coronal sections were morphed on the corresponding dark-field brain images using Euclidean transformations only, to determine the coronal plane of the section and verify area boundaries. If the brain section was non-uniformly distorted by the fixation process, special care was taken to accurately map the vicinity of the electrode tracks within the target areas. Then, green fluorescent images of the same sections were used to verify ChAT or PV expression where appropriate. VP was characterized by intermediate ChAT expression density compared to the densely labeled HDB/MCPO and the sparse and easily distinguishable CPu. The atlas images were next superimposed on red fluorescent images of the same field-of-view, which showed the DiI-labeled electrode tracks. Coordinates of electrode entry points and deepest points in the brain marked both by small electrolytic lesions and the endpoints of DiI tracks were read. These were used to interpolate the recording locations referenced to the atlas coordinate system, based on logs of the electrode descent. Based on this localization procedure, antero-posterior, lateral and dorso-ventral coordinates as well as an atlas brain area were assigned to each recording session and thus to each recorded neuron.

Immunohistochemistry

Triple immunohistochemical staining against choline acetyltranferase (ChAT), neurotensin (NT) and substance P (SP) was carried out on 50 µm coronal sections of C57Bl/6J male mice (n = 2). After washed in 0.1M phosphate buffer (PB) and tris-buffered saline (TBS), sections were incubated in blocking medium (1% human serum albumin + 0.1% Triton-X detergent) for 1 hour. Then, sections were incubated in a mixture of primary antibodies (anti-ChAT, anti-NT and anti-SP diluted in TBS, for exact concentrations and vendors, see Table 1) at 4°C for two days. After an extensive wash in TBS, the tissue was incubated in a secondary antibody solution containing Alexa 488 conjugated donkey anti-guinea pig (1:500), Alexa 594 conjugated donkey anti-goat (1:500) and Alexa 647 conjugated donkey anti-rabbit antibodies at 4°C overnight. Finally, sections were mounted on slides in Vectashield mounting medium and images were taken with a Nikon A1R confocal microscope.

Data analysis

Data analysis was carried out using custom written Matlab code (Mathworks). Action potentials were sorted into putative single neurons manually by using MClust (A.D Redish). Only neurons with good cluster quality (isolation distance > 20 and L-ratio < 0.15) were included in the final dataset for further analysis (Hangya et al., 2015; Schmitzer-Torbert et al., 2005).

After spike sorting, the activity of individual neurons was aligned to different task events (cue presentation, reward and punishment delivery). Statistics were carried out on each neuronal unit; baseline activity was defined by taking a 1 s window before the cue, then firing rate in the baseline window was compared to firing rate in the test window (0-0.5 s after the event). The one-sided hypotheses of firing rate increase and decrease were tested by Mann-Whitney U-test ($p < 0.001$; for cue-evoked activity, separately for likely reward and likely punishment cue). Neurons were sorted into different groups based on their statistically significant responses to the behaviorally relevant events (e.g. activated by cue, inhibited by reward etc.).

Autocorrelograms (ACG) were calculated at 0.5 ms resolution. *Burst index* (BI) was calculated by the normalized difference between maximum ACG for lags 0-10 ms and mean ACG for lags 180-200 ms,

where the normalizing factor was the greater of the two numbers, yielding an index between -1 and 1 (Royer et al., 2012). A neuron with a BI > 0.2 was considered to be bursting based on empirical observation reported previously (Laszlovszky et al., 2020) and confirmed by the presence of 'burst shoulders' on average ACG in the 'bursting group' and the complete lack of 'burst shoulder' on the average ACG in the 'non-bursting' group. We confirmed that the results did not depend on the choice of the BI cut-off, as using BI > 0.4 in the definition yielded similar results. To examine burst coding in the VP, analysis of neuronal responses to reinforcement-predicting cues and reinforcers were also carried out when only bursts or single spikes were considered for a neuron. A burst was detected whenever an inter-spike interval (ISI) was < 10 ms and subsequent spikes were considered as part of the burst as long as the ISI remained < 15 ms.

Characterization of rhythmic firing in the beta-gamma range was performed based on autocorrelograms. ACG peaks were detected either in the beta (16-30Hz) or gamma (30-100 Hz) frequency range. Then, the average value of a small window (±20 ms) around the peak was compared to a value calculated from a baseline period with the same algorithm. Neurons were considered rhythmically firing when this ratio was > 0.4 for the beta and > 0.25 for the gamma band. These cut-off values were determined empirically and confirmed by observing all ACGs after sorting into rhythmicity groups.

Crosscorrelograms (CCG) were calculated at 1 ms resolution. CCGs were calculated and plotted for all simultaneously recorded pairs of neurons. Synchronously activated pairs were sorted based on a significant peak exceeding the upper 95% confidence interval by at least 10 counts of co-occurrences in the CCG around zero lag. A 1-2 ms wide asymmetric peak between 1-4 ms time lags was considered a putative monosynaptic excitatory connection based on previous reports (Bartho et al., 2004; Fujisawa et al., 2008; Hangya et al., 2010). Zero-lag synchrony was not tested for pairs of neurons recorded by the same tetrodes due to potential cluster contaminations during spike sorting.

For plotting average ACG and CCG, data were Z-score normalized with their surrogate mean and standard deviation. The surrogates were generated using the shift predictor method that introduces randomized delays between the correlated signals to generate a null distribution of no correlated activity (Fujisawa et al., 2008).

It was estimated that when extracellular electrodes are advanced in the brain, within 150 µm it is theoretically possible to capture an overlapping population of neurons (Buzsáki, 2004). There is no method to unequivocally determine whether the same neuron was being recorded by extracellular electrodes on the next day (Dhawale et al., 2017); therefore, the consensus approach is to treat every session independently. Nevertheless, to test whether potential 'duplicate' recordings introduced statistical distortions that could affect results, we adapted a method from Fraser and Schwartz (Fraser and Schwartz, 2012) to determine whether the same cell was likely captured again. This method is based on the similarity of spike shape, autocorrelation and firing rates when the same neuron is recorded across sessions. The algorithm was modified to better suite tetrode microdrive recordings as opposed to Utah and Michigan arrays, as follows. Spike waveforms were normalized to the maximum on the channel with largest amplitude. Maximum waveform crosscorrelation was calculated for pairs of neurons, so that the resulting waveform similarity scores were not sensitive to small temporal shifts (Jackson and Fetz, 2007). The waveform correlations were normalized between -1 and 1 and Fisher-transformed to yield an approximate normal distribution. We calculated spike autocorrelograms (100 ms window, 5 ms resolution), and took the Fisher-transformed Pearson's correlation coefficient. The absolute log baseline firing rate difference was used as firing rate similarity measure. Unlike Fraser and Schwartz, we did not use the crosscorrelations, because neighboring cells could easily change when the electrodes were moved, rendering crosscorrelations unreliable for scoring similarity. We obtained a bootstrap null distribution from pairs of cells recorded in different mice for the three similarity scores. We used critical values corresponding to $p = 0.05$ based on the bootstrap null distributions for pairs of neurons recorded within 150 µm distance on the same tetrode.

Since we found that moderate firing rate changes could occur between and even within recording sessions, we relaxed the critical value for baseline firing rate to $p = 0.1$.

We estimated that 42 out of 704 VP recordings could be 'duplicates' of other recorded cells. We re-ran our analyses after excluding these 42 units and found that our main results did not change (Figure S10). However, since there is no gold standard method to link neuronal identity across recording days and these methods are at best considered approximate, we did not change our original dataset.

Experimental design and statistical analyses

This study includes the analysis of 704 neurons recorded from 5 mice. These sample sizes were determined according to the standards of the field and exceed the minimal requirements of most statistical tests. However, this strategy is necessary as subsequent statistics after sorting neurons into groups have group sample sizes that are not possible to plan before conducting the experiments.

Statistical comparison of central tendencies was performed using non-parametric tests (Mann-Whitney U-test for unpaired data and Wilcoxon signed rank test for paired data) as normal distribution of the underlying data could not be determined unequivocally. Distributions over categorical variables were compared by chi square test for homogeneity. The exact p-values were reported for group comparisons.

Significant firing rate changes were evaluated at $p < 0.001$ (Mann-Whitney U-test) to keep false positive rate low. Significant activation in crosscorrelograms was determined by 95% confidence intervals generated by the shift predictor method (Fujisawa et al., 2008; Kvitsiani et al., 2013). We introduced a lower bound on effect size and required >= 10 counts above this limit to disregard very small effects, which also ensures the robustness of the bootstrap process of surrogate generation. Average auto- and crosscorrelations were calculated using Z-score normalization based on a surrogate null hypothesis distribution as described above, to allow equal weighing of individual neurons in the average.

Supplemental References

Bartho, P., Hirase, H., Monconduit, L., Zugaro, M., Harris, K.D., and Buzsaki, G. (2004). Characterization of neocortical principal cells and interneurons by network interactions and extracellular features. J. Neurophysiol. *92*, 600–608.

Buzsáki, G. (2004). Large-scale recording of neuronal ensembles. Nat. Neurosci. *7*, 446–451.

Dhawale, A.K., Poddar, R., Wolff, S.B.E., Normand, V.A., Kopelowitz, E., and Ölveczky, B.P. (2017). Automated long-term recording and analysis of neural activity in behaving animals. Elife *6*, 1–40.

Fraser, G.W., and Schwartz, A.B. (2012). Recording from the same neurons chronically in motor cortex. J. Neurophysiol. *107*, 1970–1978.

Fujisawa, S., Amarasingham, A., Harrison, M.T., and Buzsáki, G. (2008). Behavior-dependent short-term assembly dynamics in the medial prefrontal cortex. Nat. Neurosci. *11*, 823–833.

Hangya, B., Li, Y., Muller, R.U., and Czurkó, A. (2010). Complementary spatial firing in place cell-interneuron pairs. J. Physiol. *588*, 4165–4175.

Hangya, B., Ranade, S.P., Lorenc, M., and Kepecs, A. (2015). Central Cholinergic Neurons Are Rapidly Recruited by Reinforcement Feedback. Cell *162*, 1155–1168.

Jackson, A., and Fetz, E.E. (2007). Compact movable microwire array for long-term chronic unit recording in cerebral cortex of primates. J. Neurophysiol. *98*, 3109–3118.

Kvitsiani, D., Ranade, S., Hangya, B., Taniguchi, H., Huang, J.Z., and Kepecs, A. (2013). Distinct behavioural and network correlates of two interneuron types in prefrontal cortex. Nature *498*, 363–366.

Laszlovszky, T., Schlingloff, D., Hegedüs, P., Freund, T.F., Gulyás, A., Kepecs, A., and Hangya, B. (2020). Distinct synchronization, cortical coupling and behavioral function of two basal forebrain cholinergic neuron types. Nat. Neurosci. *23*, 992–1003.

Paxinos, G., Franklin, K.B.J., and Franklin, K.B.J. (2001). The mouse brain in stereotaxic coordinates (Academic Press).

Royer, S., Zemelman, B. V, Losonczy, A., Kim, J., Chance, F., Magee, J.C., and Buzsáki, G. (2012). Control of timing, rate and bursts of hippocampal place cells by dendritic and somatic inhibition. Nat. Neurosci. *15*, 769–775.

Schmitzer-Torbert, N., Jackson, J., Henze, D., Harris, K., and Redish, a D. (2005). Quantitative measures of cluster quality for use in extracellular recordings. Neuroscience *131*, 1–11.

Solari, N., Sviatkó, K., Laszlovszky, T., Hegedüs, P., and Hangya, B. (2018). Open Source Tools for Temporally Controlled Rodent Behavior Suitable for Electrophysiology and Optogenetic Manipulations. Front. Syst. Neurosci. *12*, 18.
